# Editorial: AI-driven scientific discovery—transforming research across disciplines

**DOI:** 10.3389/frai.2026.1908389

**Published:** 2026-07-20

**Authors:** Thomas Hartung, Kemal Oflazer, Paolo Napoletano

**Affiliations:** 1 Center for Alternatives to Animal Testing (CAAT), Department of Environmental Health and Engineering, Bloomberg School of Public Health and Whiting School of Engineering, Johns Hopkins University, Baltimore, MD, United States; 2 Center for Alternatives to Animal Testing-Europe, University of Konstanz, Konstanz, Germany; 3 Language Technologies Institute, Carnegie Mellon University, Pittsburgh, PA, United States; 4 Department of Informatics, Systems and Communication, University of Milano-Bicocca, Milan, Italy

**Keywords:** agentic AI, artificial intelligence, interdisciplinary research, laboratory automation, large language models, machine learning, responsible AI, scientific discovery

## A field in transition

When the World Economic Forum named “*AI for scientific discovery*” among its Top 10 Emerging Technologies of 2024, it formalized a shift that practicing researchers had already begun to feel (World Economic Forum, 2024). Across the natural, biomedical, and social sciences, artificial intelligence has moved from a specialist's instrument to a general-purpose engine of inquiry, one that predicts molecular structures, mines the literature, forecasts markets, guards networks, and, increasingly, acts on its own conclusions. What unites these developments is not a single algorithm but a shared methodological turn: a willingness to let learned models, rather than hand-built rules, find structure in data too vast or too noisy for unaided human pattern recognition.

Three forces give the moment its distinctive character. First, the maturation of deep learning has made high-dimensional, non-linear prediction routine in domains such as cardiology, agronomy, aquaculture, cybersecurity, that share little except the shape of their data. Second, large language models have collapsed the distance between a researcher's question and a working analytic pipeline, reshaping how science is conceived, conducted, and communicated. Third, and most consequentially, AI is becoming increasingly agentic: multimodal systems are beginning to interpret knowledge, orchestrate software, and interact with physical laboratory hardware within human-designed workflows and experimental constraints. Each advance enlarges what is possible while sharpening enduring questions of reproducibility, auditability, bias, safety, and equitable access.

This Research Topic was conceived to capture that breadth honestly. We invited contributions spanning molecular and structural biology, AI-enhanced research processes, healthcare, applied modeling, the language sciences, and the ethical, legal, and practical questions that follow. The eight articles gathered here (five original research studies and three reviews) were contributed by authors working across four continents and drew close to 50,000 views. They do not exhaust the field. Together, however, they offer an unusually candid cross-section of where AI is genuinely changing practice and where the slower work of validation and governance remains to be done.

## The changing practice of science itself

Two reviews map the meta-level transformation, how AI is reshaping the conduct of research rather than any single result. Hartung's recent article “*AI, agentic models and lab automation for scientific discovery—the beginning of scAInce*” argues that science has entered an era of multimodal, agentic systems that listen, see, speak, and act. Merging the author's 2024 World Economic Forum white paper with advances through mid-2025, Hartung charts a course from automated literature synthesis and hypothesis generation to self-driving laboratories, organoid intelligence, and climate-scale forecasting. The central thesis is a “*co-pilot to lab-pilot*” transition in which AI moves beyond interpretation toward the coordination of experimental and computational workflows. This shift promises substantial efficiency gains, while amplifying concerns about reproducibility, auditability, safety, and equitable access. The article situates within emerging governance regimes such as the EU AI Act and ISO 42001.

Where Hartung looks at the laboratory, Ng looks at the desk. “*A structured framework for effective and responsible generative artificial intelligence chatbot prompt engineering throughout the scientific process*” offers health and medical researchers a 10-chapter, evidence-informed guide to using large-language-model chatbots well. It walks prompt engineering through the entire research cycle: question development, study design, literature searching, selection of reporting guidelines and appraisal tools, quantitative and qualitative analysis, writing, dissemination, and implementation. At the same time, the author emphasizes that generated content should be treated as provisional, verified against credible sources, and held to disciplinary standards. Its sober treatment of hallucination, embedded bias, transparency, and accountability makes the same point, Hartung does from the opposite end of the workflow: AI should augment, not replace, human expertise.

## Health and biomedicine

Two contributions show AI compressing biomedical timelines. Elfatimi et al.'s review, “*Artificial intelligence and machine learning in the development of vaccines and immunotherapeutics—yesterday, today, and tomorrow*,” surveys how AI and deep learning are displacing trial-and-error experimentation and costly *in vivo* testing. By integrating computational models, systems vaccinology, and multi-omics data, these methods refine B- and T-cell antigen and epitope selection, sharpen patient phenotyping, and deepen understanding of immune regulation and evasion. The authors point toward AI's potential to reduce reliance on, and in selected contexts replace, animal-based preclinical testing, echoing recent United States NIH and FDA proposals, and to support precision, personalized vaccines and immunotherapeutics for infectious diseases and cancers.

Kahraman's original research, “*Machine learning techniques for improved prediction of cardiovascular diseases using integrated healthcare data*,” demonstrates the value of data integration for clinical decision support. After merging multiple public healthcare datasets on shared features into a cleaned corpus of roughly 311,710 records, the study benchmarks several classifiers, with the CatBoost model reaching the highest area under the curve at 94.1%, that is, evidence that gradient-boosted models on harmonized, large-scale clinical data can support earlier, more reliable cardiovascular risk assessment.

## AI across applied domains

Three studies carry the same machine-learning toolkit into strikingly different settings. Zhang et al. “*Quantification of feeding intensity and feeding control of largemouth bass based on water surface vibration characteristics*” turns precision aquaculture into a closed-loop control problem. By quantifying triaxial water-surface vibration signals and feeding them to a Long Short-Term Memory network, the authors predict feeding intensity with high fidelity (*R*^2^ = 0.883), outperforming GRU and Transformer baselines. Deployed on a sub-$200 embedded system, the model achieves residual feed rates of 0.8% or less, surpassing optical-flow and graph-neural-network approaches at a fraction of their computational cost, and exemplifying the move from prediction to autonomous action.

Sanwa et al. address agricultural economics in “*Forecasting global monthly cotton prices: the superiority of NNAR models over traditional models*.” Systematically comparing classical statistical methods (ARIMA, ETS, STL, TBATS, and Theta) with a Neural Network Auto-Regressive model and hybrids, they find the NNAR specification most accurate, with a mean absolute percentage error of just 1.19%. Its 30-month forecast points to cyclical fluctuation between roughly $0.66 and $0.74 per pound, an illustration of how learned models capture the non-linear dynamics that defeat conventional forecasts, with direct value for farmers, traders, and policymakers.

Akinbowale et al. turns to the security of the connected world in “*Machine learning based approach to intrusion detection in internet of things environments*.” Training Decision Tree, Random Forest, and Support Vector Machine classifiers on more than a million labeled flow records spanning 34 attack types, they find tree-based models both accurate and interpretable: the Decision Tree reached 99.36% accuracy, narrowly ahead of Random Forest at 99.27%, while the SVM trailed at 80.08% under the weight of the data. Feature-importance analysis singled out inter-arrival time and total packet size as the key discriminators of malicious behavior, pointing toward lightweight, real-time defenses for resource-constrained IoT networks.

## Language, culture, and inclusion

A final study widens the lens from performance to fairness. Khalilia et al. confronts a rarely discussed bias in the lexical-semantic resources that underpin natural language processing *in* “*Crowdsourcing lexical diversity*”: their tilt toward English and Anglo-Saxon culture, which erases language-specific concepts and untranslatable “*lexical gaps*.” Their LingoGap platform uses crowdsourced microtasks to compare lexemes across languages, identifying equivalent terms, language-specific terms, and gaps. Applied to food terminology in English–Arabic and Standard Indonesian–Banjarese, their method surfaced 2,140 and 951 lexical gaps, respectively, that is, a scalable route to more culturally faithful resources, and a reminder that the data on which AI is built encodes the worldviews of those who assemble it.

## What these articles contribute together

Read as a Research Topic, these eight contributions amount to more than a sampler of applications. Four threads bind them. The first is methodological convergence: the same families of models (gradient-boosted trees, recurrent and attention-based networks, neural auto-regression, and large language models) recur from cardiology to cotton markets, from fish tanks to firewalls. AI has become a methodological *lingua franca*, and much of the field's momentum now comes from transferring techniques across disciplinary boundaries rather than inventing them anew.

The second thread is the migration from interpretation to action. Hartung's self-driving laboratories and Zhang's closed-loop feeding controller are different scales of the same trajectory: systems that do not merely analyze data but change the world based on their analysis. This is where the promise of efficiency is greatest and where the governance stakes rise most steeply. The third thread is precisely that insistence on responsibility: Ng's demand for verification and transparency, Hartung's grounding in the EU AI Act and ISO 42001, and Khalilia's attack on embedded bias all converge on a shared conviction that human judgment, auditability, and fairness must scale alongside capability.

The fourth thread is inclusivity in the widest sense—of disciplines, of geographies, and of subjects of research. The authorship spans four continents; the applications reach from high-income clinical systems to smallholder agriculture and under-resourced languages; and the biomedical contributions point toward replacing animal testing with computational models, extending the reach of ethical research practice. Taken together, the Research Topic supports a measured conclusion: AI is genuinely transforming scientific discovery across disciplines, but its value is realized only when predictive power is matched by rigorous validation, transparent reporting, and attention to whom the technology serves. Across these contributions, AI emerges not as a substitute for scientific judgment, but as a force reshaping where such judgment is most needed: in problem formulation, validation, interpretation, and governance. We thank the authors, reviewers, and readers who made this Research Topic possible, and we offer it as a platform for the interdisciplinary dialogue that the next phase of AI-driven discovery will demand.
